# Fungal Dysbiosis Correlates with the Development of Tumor-Induced Cachexia in Mice

**DOI:** 10.3390/jof6040364

**Published:** 2020-12-13

**Authors:** Daniela L. Jabes, Yara N. L. F. de Maria, David Aciole Barbosa, Kaltinaitis B. N. H. Santos, Lucas M. Carvalho, Ana Carolina Humberto, Valquíria C. Alencar, Regina Costa de Oliveira, Miguel L. Batista, Fabiano B. Menegidio, Luiz R. Nunes

**Affiliations:** 1 Núcleo Integrado de Biotecnologia, Universidade de Mogi das Cruzes (UMC), São Paulo 08780-911, Brazil; 11152500497@alunos.umc.br (Y.N.L.F.d.M.); 11121503188@alunos.umc.br (D.A.B.); 11101100049@alunos.umc.br (K.B.N.H.S.); 11171502445@alunos.umc.br (L.M.C.); 11191100818@alunos.umc.br (A.C.H.); valquiria.alencar@ufabc.edu.br (V.C.A.); reginaco@umc.br (R.C.d.O.); miguelj@umc.br (M.L.B.J.); fabianomenegidio@umc.br (F.B.M.); 2 Centro de Ciências Naturais e Humanas, Universidade Federal do ABC (UFABC), São Bernardo do Campo 09606-045, Brazil

**Keywords:** microbiome, microbiota, mycobiota, cachexia, next generation sequencing, NGS

## Abstract

Cachexia (CC) is a devastating metabolic syndrome associated with a series of underlying diseases that greatly affects life quality and expectancy among cancer patients. Studies involving mouse models, in which CC was induced through inoculation with tumor cells, originally suggested the existence of a direct correlation between the development of this syndrome and changes in the relative proportions of several bacterial groups present in the digestive tract. However, these analyses have focus solely on the characterization of bacterial dysbiosis, ignoring the possible existence of changes in the relative populations of fungi, during the development of CC. Thus, the present study sought to expand such analyses, by characterizing changes that occur in the gut fungal population (mycobiota) of mice, during the development of cancer-induced cachexia. Our results confirm that cachectic animals, submitted to Lewis lung carcinoma (LLC) transplantation, display significant differences in their gut mycobiota, when compared to healthy controls. Moreover, identification of dysbiotic fungi showed remarkable consistency across successive levels of taxonomic hierarchy. Many of these fungi have also been associated with dysbioses observed in a series of gut inflammatory diseases, such as obesity, colorectal cancer (CRC), myalgic encephalomyelitis (ME) and inflammatory bowel disease (IBD). Nonetheless, the dysbiosis verified in the LLC model of cancer cachexia seems to be unique, presenting features observed in both obesity (reduced proportion of Mucoromycota) and CRC/ME/IBD (increased proportions of Sordariomycetes, Saccharomycetaceae and *Malassezia*). One species of Mucoromycota (*Rhyzopus oryzae*) stands out as a promising probiotic candidate in adjuvant therapies, aimed at treating and/or preventing the development of CC.

## 1. Introduction

Cachexia (CC) is recognized as a metabolic syndrome associated with several underlying diseases, such as cancer, chronic kidney disease, and chronic heart disease, among others [1]. It is characterized by the reduction of muscle mass, depletion of body fat and generalized chronic inflammation [1]. Its prevalence among patients with different types of cancer contributes to significantly decrease their life quality and expectancy, being an important cause of morbidity/mortality in more than 80% of advanced cancer cases and accounting for more than 20% of deaths [2]. Until the 1980s, cachexia was attributed to anorexia and increased energy expenditure. However, enteral or para-enteral administration of nutritional supplements is not sufficient to reverse cachexia symptoms, refuting the hypothesis that nutrient deficiency is the main causative agent of this syndrome. During the early 1990s, cachexia came to be defined as a chronic inflammatory syndrome, involving metabolic changes derived from factors produced by both external agents (such as tumors), as well as by the affected organism [1].

Studies aimed at characterizing the microbiota of mice, during the development of cancer-induced cachexia, revealed significant alterations in the composition of their gut bacterial population (dysbiosis), when compared to healthy control animals [3,4]. The main characteristic of such dysbiosis involves an increased proportion of Enterobacteriaceae in CC-affected animals—a phenomenon also observed during the development of several diseases and syndromes associated with gut inflammation, such as gastric cancer [5], diabetes [6], obesity [7], and inflammatory bowel diseases (IBD), such as ulcerative colitis (UC) and Crohn’s disease (CD) [8], among others. More recently, Pötgens et al. (2018) [9] cultivated fecal material from cachectic mice in selective coliform media, identifying *Klebsiella oxytoca* as the main representative of Enterobacteriaceae present in stool samples obtained from these animals. Additional studies led these authors to suggest that *K. oxytoca* could represent an intestinal pathobiont, capable of affecting both thickness and permeability of the mucus barrier that protects the digestive epithelium of mice, when over-represented in their gastrointestinal tract [9].

These findings demonstrated that gut dysbiosis could be an important factor for the development of cancer-induced cachexia and suggested that the control of gut microbiota composition, through the use of specific antibiotics, prebiotics and probiotics could be used as an adjuvant approach to treat and/or prevent the development of cachexia. However, all CC studies carried out so far have remained focused solely on analyzing the bacterial microbiota, ignoring the possible existence of changes in the relative populations of fungi during the development of this syndrome. Thus, the present study sought to expand the scope of such analyses by characterizing the main alterations verified in the fungal population (mycobiota) present in the guts of mice, after CC induction by subcutaneous inoculation with Lewis lung carcinoma (LLC) cells (a model widely employed for the study of CC [10,11]. In general, it was possible to verify the existence of significant differences involving the gut mycobiota of CC animals, in comparison to healthy controls. The fungal dysbiosis of the cachectic animals revealed alterations in the relative proportions of several fungal taxa, consistently distributed in successive levels of taxonomic hierarchy. In many cases, relative changes in these populations have also been described in dysbioses associated with other gut inflammatory diseases, although the CC-associated dysbiosis seems to display a unique signature. Moreover, some of the microorganisms found to be expanded among CC-affected animals display metabolic characteristics that may directly contribute to the development of cachexia. Finally, we identified fungal species preferably expanded in the gut of healthy animals, which can be grown axenically, under laboratory conditions. These latter microorganisms represent natural candidates to be tested as probiotics in adjuvant therapies, aiming at the treatment and/or prevention of cachexia.

## 2. Materials and Methods

### 2.1. Cachexia Induction in C57BL/6 Mice and Collection of Stool Samples for Characterization of the Gut Mycobiota during the Development of Cachexia

The experiments described herein were conducted with 6–8-week old male mice, weighting ~17 to 24 g (22.7 g, on average). Animals were selected from colonies of C57BL/6 mice (Jackson Laboratory), which were bred and kept in plastic cages, in a rodent-only controlled environment, with low levels of external noise, 24 ± 1 °C average temperature and light/dark cycles every 12 h. Food (Nuvilab CR1^®^—Nuvital) and water were provided ad libitum. All animals were individually marked with identifying rings at the beginning of the experiment. Gut microbiota of animals used in this study was synchronized as suggested in Macpherson and McCoy (2015) and Franklin and Ericsson (2017) [12,13]. Briefly, two weeks before beginning the experimental procedures, 4–6-week old animals were distributed in 5 plastic cages (4 animals per cage) and rotated among the cages every 2 days. Each group was kept in contact with shavings and drippings from the previous group for 24 h, before being transferred to a fresh and sterile cage. After this period, a group of 10 animals was randomly chosen as a control group (CT), having received only subcutaneous injection with 200 μL saline solution in their right flank. The others (20) were similarly inoculated with 3.5 × 10^5^ LLC tumor cells (suspended in 200 μL of Dulbecco’s modified Eagle’s medium). Next, the animals were transferred to sterile cages (2 animals per cage) and kept, under the conditions described above, for a period of 28 days, when cachexia symptoms could be verified [14,15]. Stool samples were collected individually, by gently pulling up the animals’ tails and guiding them into 50-mL sterile Falcon tubes, for a few seconds. Next, stool samples were properly identified, frozen in liquid N_2_ and stored at −70 °C. The genetic material obtained from the samples collected at day 28 was used to characterize the fecal microbiota of both CT and LLC-inoculated animals, as described below. All animals were sacrificed by decapitation, without anesthesia, immediately after collecting the last stool samples. Blood, organ, and tissue samples from these mice were harvested and frozen at −70 °C. This experimental design was evaluated and approved by the Ethics Committee for the Use of Animals in Research, at the University of Mogi das Cruzes (CEUA-UMC), under permission code n: 012/2017, issued on Aug/01/2017.

### 2.2. Assessment of Cachexia Progression

After inoculum, either with saline solution or LLC cells, all animals were weighed daily, on a precision scale (always at the same time), throughout the entire course of the experiment. This information was used to calculate the Cachexia Index (CI) in the LLC-inoculated animals, following the methodology described in Voltarelli et al. (2017) [15]. According to these calculations, 8 animals showed CI values ≥ 5, indicating full development of cachexia [15]. These animals were further evaluated for their cachectic condition by comparing the relative weight of the gastrocnemius muscle and by histometric characterization of adipose tissue (both collected and evaluated at day 28). Briefly, relative gastrocnemius mass was obtained, for each animal, by weighting the whole muscle and dividing their values by final animal weight (tumor subtracted, in the case of CC animals). For adipose tissue evaluation, samples from the epididymal adipose tissue were prepared and stained with hematoxylin-eosin (HE) and digital cross-section images of the slides were obtained, using a Leica optical microscope (model DMLP), coupled with an LG CCD camera (model GC-415-MD). In total, 5 images were captured per animal and average adipocyte area was calculated from 500 randomly selected adipocytes/animal, with the aid of the ImagePro-Plus 6.0 software (Media Cybernetics, Silver Spring, MD, USA), as previously described [16,17]. Finally, expression of molecular markers for muscular atrophy (Atrogin) and inflammation (IL6 receptor, involved in the activation of pro-inflammatory responses by the trans-signaling IL-6 pathway [18]) were obtained by real-time quantitative PCR (qPCR) (see [19,20] for details). These 8 animals were designated Cachectic Group, or CC, which was further used for subsequent microbiome analyses. A similar group of 8 animals (the Saline Control Group, or SC) was selected among the CT animals, to establish a control group of similar size for future comparisons with the CC group.

### 2.3. Sample Processing, Sequencing, and Analysis of ITS1 Amplicon Libraries

The extraction of fungal DNA from stool samples was performed with the aid of the DNeasy Power Soil kit, according to the manufacturer’s instructions (Qiagen, Hilden, Germany). The material from these extractions was used to amplify sequences from the internal spacer region 1 (ITS1), as described by Mallick et al. (2017) [21]. The complete protocol employed to prepare and sequence the ITS1 libraries can be accessed from the Open Science Framework (OSF) repository (https://osf.io/). Raw sequences have been deposited at OSF (https://osf.io/5fxgn) and SRA repository (https://www.ncbi.nlm.nih.gov/sra), under Submission ID PRJNA642649. ITS1 amplicon sequences were processed by a custom pipeline specifically developed at our laboratory, which is available from a Jupyter Notebook file, in a Docker container available at: https://osf.io/5fxgn. Briefly, individual paired-ended reads were joined and the resulting amplicons were checked for quality and trimmed. Amplicon sequences were next used to construct an operational taxonomic unit (OTU) Table by the open OTU picking strategy. The resulting OTU Table was filtered to keep only OTUs with abundance above 0.00001 and used to calculate the absolute number of fungi (alpha diversity) present in the stool samples from SC and CC animals, at different taxonomic levels. For beta diversity analyses, the OTU Table was further filtered to present only OTUs present in at least 70% of the animals within each group, minimizing the effect of taxa over-represented in few individuals within a group, but not consistently present throughout the group. A thorough description of these processes can also be found at OSF (https://osf.io/b4wvd/).

### 2.4. Fungal Population and Diversity Analysis of the Gut Mycobiota from SC and CC Mice

Measurement of population ratios and Alpha/Beta diversity analyses were conducted with the aid of “MicrobiomeAnalyst” [22], using the OTU Table derived from the analytical steps mentioned above. To load the data, the Low Count Filter was adjusted to minimum values (Minimum Count = 0 and Prevalence in Sample = 10) and the OTU Table was rarefied by the number of sequences in the smallest library (Rarefy to the Minimum Library Size). All other parameters remained unchanged. Statistical significance of differences involving alpha diversity measurements between SC and CC groups was evaluated by a Mann–Whitney U Test (*p* ≤ 0.05 as threshold). Variations in beta diversity were conducted with the aid of Bray–Curtis metrics and visualized by a non-metric multidimensional scaling (NMDS) algorithm, followed by statistical validation with PERMANOVA (*p* ≤ 0.05 as threshold). All these analyses were conducted using MicrobiomeAnalyst. Fungal population ratios were calculated and statistically validated as described in Coker et al. [23], using relative population composition obtained from MicrobiomeAnalyst.

### 2.5. Identification of Differentially Represented Taxa in SC and CC Mice, Using Linear Discriminant Analysis Effect Size (LEfSe)

Initially, core microbial populations were determined for each group separately (SC and CC), using the QIIME command “compute_core_microbiome.py” to select only OTUs present in ≥70% of the animals in the group. The OTU Tables containing the SC and CC Core 70 microbiotas were then integrated, using the QIIME “merge_otu_tables.py” command, resulting in a Core 70 OTU Table for the entire SC X CC dataset. The use of 70% as a threshold to define Core microbiotas followed other studies described in the literature, where such limits vary between 50% and 80% [24,25,26,27,28,29,30,31,32,33]. Next, the Core 70 OTU Table was submitted to the QIIME “summarize_taxa.py” script, integrating the results obtained for each taxon and converting their absolute prevalence into relative ratios. This relative OTU Table was then submitted to a linear discriminant analysis effect size (LEfSe) evaluation, using the default settings of the Galaxy LEfSe tool, available at https://huttenhower.sph.harvard.edu/galaxy/.

### 2.6. Quantitative Real-Time PCR (qPCR)

Equimolar samples of DNA extracted from the stool samples from each mouse (the same DNAs used for the construction of amplicon libraries) were consolidated into two pools, representing the SC and CC groups. Samples of these pools were further purified, with the aid of the DNeasy Power Soil kit (Qiagen) and used in qPCR experiments, with the aid of primers previously described in the literature as specific to the genera *Saccharomyces*, *Kazachstania,* and *Malassezia*, as well as to the species *Saccharomyces cerevisiae*, *Malassezia dermatis*, *Malassezia japonica*, *Rhizopus oryzae,* and *Penicillium citrinum* [34,35,36,37,38,39,40,41]. The Ct values obtained for each of these taxa were normalized by the Ct obtained with qPCR amplifications, using the primer pair used to build the fungal amplicon libraries.

## 3. Results

### 3.1. Cachexia Induction and Characterization in Mice

**Figure 1:**
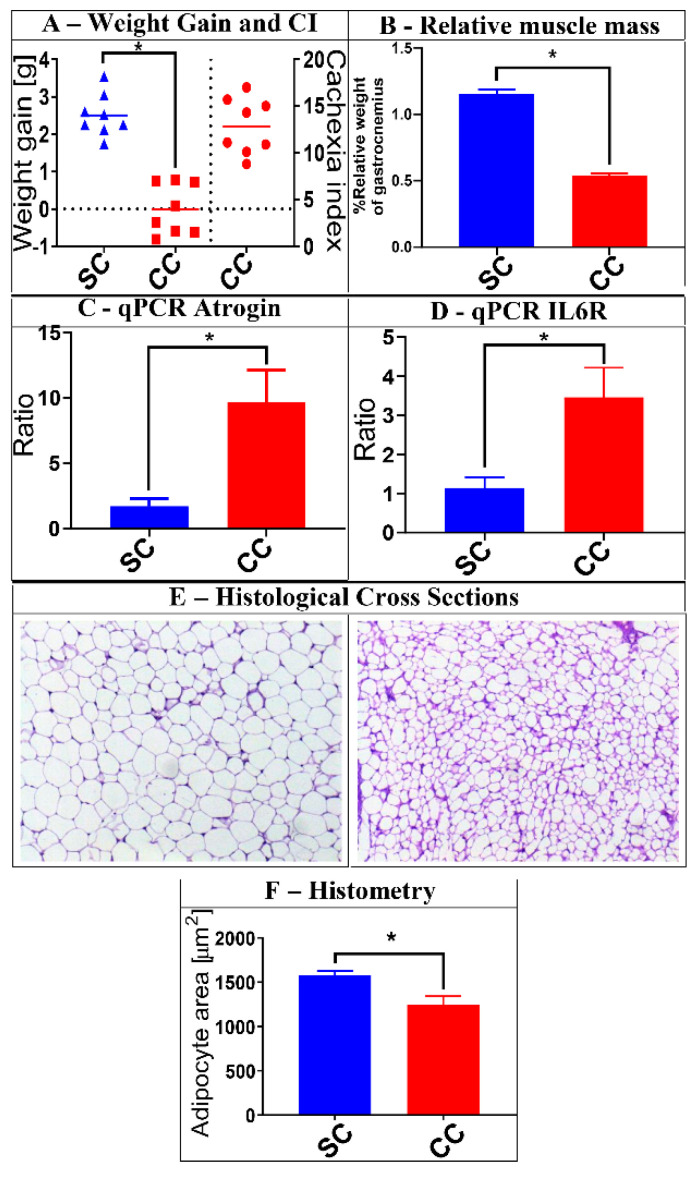
Characterization of cachexia in the animals selected for this study. A group of 16 animals was selected for the analyses described herein. Eight (8) of them received saline injection (SC group), while the other 8 were injected with Lewis lung carcinoma (LLC) cells (CC group). After 28 days, the animals were evaluated for a series of characteristics typically associated with cachexia. Panel (**A**) shows, on the left part of the graph, the average weight gain of the SC and CC animals 28 days after injection (tumor weight subtracted), as well as the Cachexia Indexes (CI) among CC animals (on the right part of the graph) (Weight gain: SC = 2.508 ± 0.1996; CC = −0.00625 ± 0.2391. Cachexia index: CC = 12.86 ± 1.06). Panel (**B**) shows the reduced relative mass of gastrocnemius muscle in CC animals, when compared to SC controls (SC = 1.151 ± 0.09864; CC = 0.5373 ± 0.04663). Panels (**C**,**D**) show that CC animals also display increased relative expression of muscular atrophy and inflammation markers (Atrogin and IL6R, respectively), as verified by qPCR (Atrogin: SC = 1.421 ± 0.3972; CC = 9.661 ± 2.471. IL6R: SC = 1.062 ± 0.1726; CC = 3.573 ± 0.707). Panel (**E**) shows examples of histological analyses made with epididymal adipose tissue obtained from SC (left) and CC (right) animals, demonstrating significant reduction in the average adipocyte area in CC animals. These reductions are confirmed by histometric evaluation of representative cross sections obtained from all animals within each group (Panel (**F**)) (Adipocyte area: SC = 1573 ± 45.83; CC = 1197 ± 77.71). Magnification of adipose tissue: 20×; * = *p* ≤ 0.05, after Mann–Whitney U test. Results for SC animals are shown in blue, while results for CC animals are shown in red. Plots represent mean and mean standard errors.

A group of 20 mice were submitted to LLC tumor cell transplantation and monitored for a 28-day period, under controlled conditions, as described in Materials and Methods. At the end of this period, animals were sacrificed, and their weight gain was compared to a group of 10 control animals (CT group), to determine their respective Cachexia Indexes (CI) (Figure 1). Eight (8) of these animals displayed CI ≥5 and were classified as the CC (cachectic) group. Accordingly, we selected a group of equal size, among the CT animals, to constitute the Saline Control (SC) group, which were used for further comparisons with the CC mice. As observed in Figure 1, animals from the CC group displayed a series of additional characteristics of cachexia, when compared to the SC mice, including reduced relative mass of gastrocnemius muscle, morphometric changes in adipose tissue (decreased adipocyte area), and increased expression of marker genes for muscular atrophy and inflammation (Atrogin and IL6R, respectively) [16,20]. Thus, stool samples obtained from SC and CC mice, at day 28, were further processed to characterize their fungal gut populations (mycobiota).

### 3.2. Characterization and Comparison of Fungal Gut Populations (Mycobiota) in SC and CC Animals

Amplicon libraries referring to the ITS1 sequences were built for the animals of both SC and CC groups, as described in Materials and Methods and subjected to sequencing in an Illumina MiSeq DNA sequencer. The number of amplicons obtained for each animal, after all pre-processing steps, varied between 66,436 and 163,377, as shown in Table A1. These sequences were submitted to an analytical pipeline, specially developed in our laboratory, generating a table of operational taxonomic units (OTU Table) that was later used to analyze and compare the fungal gut populations (mycobiota) of SC and CC animals.

**Figure 2:**
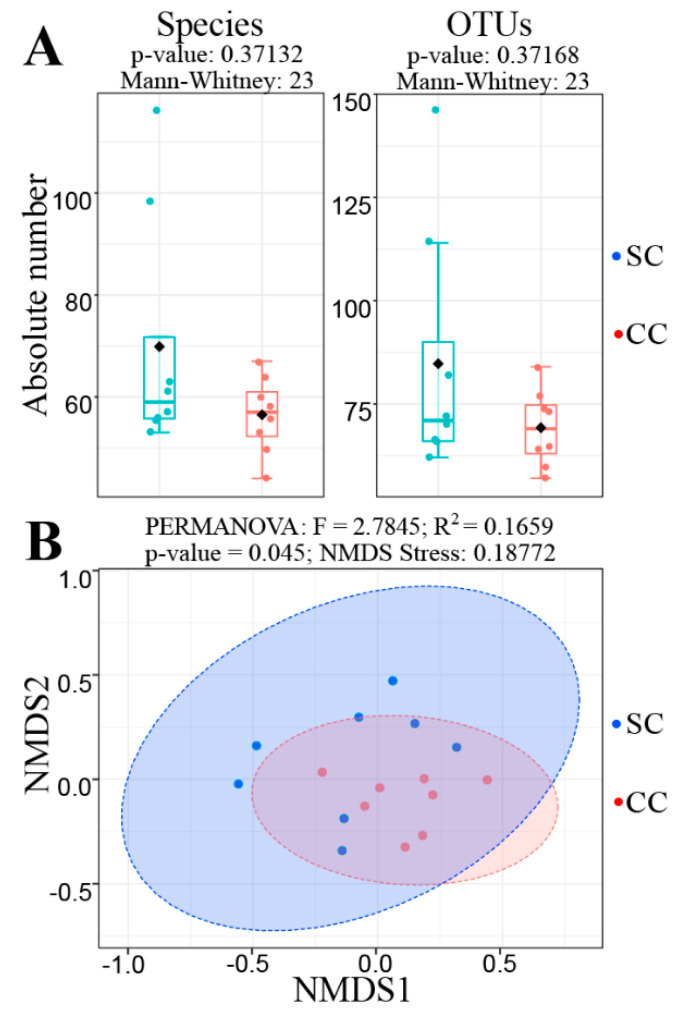
Alpha and Beta diversity analyses comparing the mycobiota of SC and CC animals. Panel (**A**) shows the result of an alpha diversity analysis, displaying the absolute number of fungi obtained for the levels of species (left) and operational taxonomic units (OTU) (right), in both SC and CC animals. In both cases, it was not possible to verify significant changes in fungal alpha diversity between the two groups, since *p*-values obtained from such analyses, after a Mann–Whitney U test, were always above 0.05. Panel (**B**) shows the result of a Beta-diversity analysis to evaluate differences in composition between the fungal populations present in the stool samples from SC and CC animals. This analysis was performed with the aid of a non-metric multidimensional scaling (NMDS) algorithm (based on the Bray–Curtis index), using the same OTU Table described above. The NMDS analysis shows that differences in mycobiota composition allow statistically significant discrimination between the SC and CC groups (at *p* = 0.045, as verified by PERMANOVA), although the two main components of the NMDS analysis do not allow us to visualize such distinction. Animals from the SC group are shown in blue, while animals from the CC group are shown in red.

Surprisingly, initial analyses revealed that there was no alteration in fungal alpha-diversity when the fecal mycobiota of SC and CC animals were compared, since the absolute number of fungal species and OTUs did not show statistically significant differences (Figure 2A). Similar results were observed when using alternative alpha-diversity metrics (Shannon, Chao1, ACE, Simpson and Fisher) and/or after extending the analyses to other taxonomy levels (phylum to genus) (not shown). However, a beta diversity analysis revealed that the SC and CC animals display sufficient differences in the composition of their gut mycobiota to discriminate the two groups (Figure 2B). This analysis was performed with the aid of a non-metric multidimensional scaling (NMDS) algorithm, using the Bray–Curtis index to compare the SC and CC mycobiota contents. The result from this analysis was subsequently evaluated by PERMANOVA, confirming that the SC and CC groups exhibit sufficient differences in the composition of their gut fungal populations to allow their discrimination at statistically significant levels (*p* = 0.045), although the two main components of the NMDS analysis do not provide visual separation between the two groups (Figure 2B).

**Figure 3:**
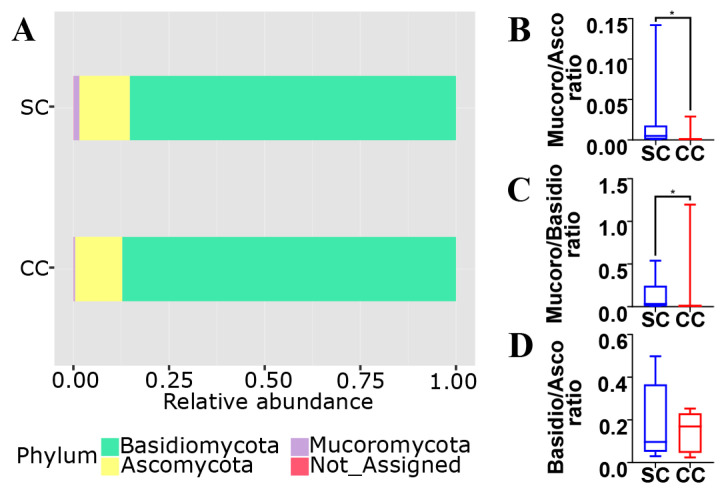
Phylum composition of the mycobiota from SC and CC animals. Panel (**A**) shows that the gut mycobiota of SC animals are mostly represented by Ascomycota (~85.1%), followed by Basidiomycota (~13.3%) and Mucoromycota (~1.5%). These proportions are shifted in the gut mycobiota of CC animals to ~87.2% (Ascomycota), ~12.5% (Basidiomycota), and ~0.4% (Mucoromycota). These values were used to calculate the specific ratios involving the main fungal phyla, between SC and CC animals: Mucoromycota/Ascomycota (Panel (**B**)), Mucoromycota/Basidiomycota (Panel (**C**)) and Basidiomycota/Ascomycota (Panel (**D**)). Results for SC animals are shown in blue, while results for CC animals are shown in red (* = *p* ≤ 0.05, after Mann–Whitney U test).

As observed in Figure 3A, the vast majority of the fungal population in the gut of both SC and CC animals involves members from the phyla Ascomycota (~85 to 87%), Basidiomycota (~12 to 13%) and Mucoromycota (~0.4 to 1.5%). When the two groups are compared, however, it is possible to verify that CC animals display statistically significant reduction (~16×) in both Mucoromycota/Ascomycota and Mucoromycota/Basidiomycota ratios (Figure 3B,C). It is also possible to observe a slight increase (~1.7×) in the Ascomycota/Basidiomycota ratio of CC animals, although this difference did not display statistical significance under our experimental conditions (Figure 3D). Interestingly, similar population imbalances involving the main fungal phyla have also been observed in association with alternative gut inflammatory diseases and disorders (see below). Unfortunately, it was not possible to detect statistically significant alterations involving other groups of fungi below the level of phylum, probably due to limitations involving the use of classical univariate statistical inference methods on microbiota compositional data (see [21]).

**Figure 4:**
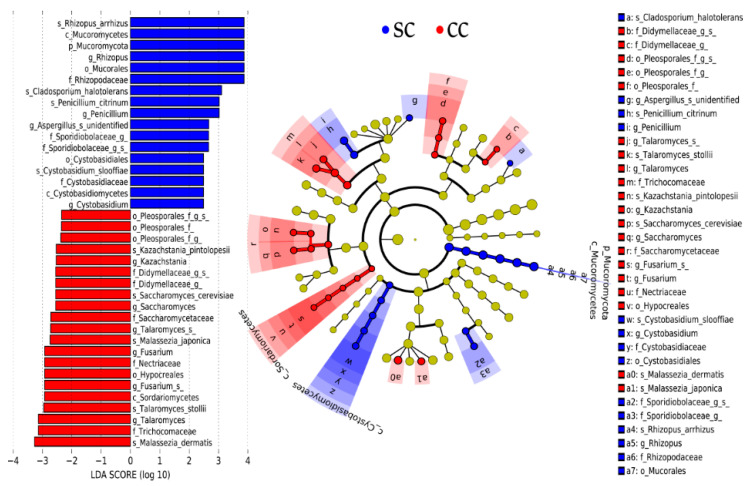
Identification of the main fungal taxa differentially represented in stool samples from SC and CC animals. The Core 70 OTU Table (see Methods) was subjected to a linear discriminant analysis effect size (LEfSe) analysis to identify differentially represented taxa between CC and SC animals. Taxa preferentially present in CC animals displayed Linear Discriminant Analysis (LDA) Scores ≤2, while taxa preferentially present in SC animals displayed LDA Scores ≥2. All taxa identified in this analysis display statistical significance at *p* ≤ 0.05, which was used as a threshold in the LEfSe analysis. All differentially represented taxa (at different taxonomic levels) are shown in the left panel of the figure and their distribution in a phylogenetic dendrogram is shown at the center panel. The concentric circles of the dendrogram show the taxonomic hierarchy, from phylum (innermost circle) to species (outermost circle) and the different microorganisms distributed in each node can be identified with the aid of the legend shown on the right panel. Microorganisms overrepresented in stool samples from SC animals are shown in blue, while microorganisms overrepresented in stool samples from CC animals are shown in red.

To overcome such limitations, the mycobiota of SC and CC animals were further compared through LEfSe (linear discriminant analysis effect size), a widely used tool specifically developed to identify differentially represented elements from microbiome data [42] (Figure 4). This analysis was conducted with a Core 70 OTU Table, containing only OTUs present in, at least, 70% of the animals in each group. This strategy is often used to reduce effects related to individual variability among subjects, minimizing the effect of taxa that are over-represented in few individuals within a group, but not consistently present throughout the group [24,25,26,27,28,29,30,31,32,33]. As expected, LEfSe analysis confirmed that members of Mucoromycota were present in higher proportion in the stool samples of SC animals, when compared to CC. Moreover, the SC-overrepresented Mucoromycota identified by LEfSe displayed remarkable consistency across all hierarchical levels of fungal taxonomy, involving representatives from the Mucoromycetes class, Mucorales order and Rhizopodaceae family, with emphasis on the species *Rhizopus arrhizus*, also classified as *R. oryzae* [43].

LEfSe also confirmed that there was no significant alteration in the overall distribution of Ascomycota and Basidiomycota phyla between SC and CC animals, but detected a series of fungal groups belonging to these two phyla that were differentially distributed in the stool samples from both SC and CC groups (Figure 4). Interestingly, the fungal groups that displayed the most consistent distribution across successive hierarchical levels of taxonomy highlight a series of taxa (mostly Ascomycota) that seem to be preferably expanded among CC animals (Figure 4). These include, for example, representatives from all taxonomic levels of the Sordariomycetes class, including members of the Hypocreales order and Nectiriaceae family, with emphasis on a species of *Fusarium*, with unspecified classification (wuc). Members of the Saccharomycetaceae family also showed population expansion in the stool samples of cachectic animals, including representatives of *Saccharomyces* and *Kazachstania* (with emphasis on *Saccharomyces cerevisiae* and *Kazachstania pintolopesii*, respectively). Two representatives of *Talaromyces* (*Talaromyces stollii* and another species (wuc)) were also identified as preferentially present in the feces of cachectic mice, when compared with SC animals. Additional Ascomycota displaying population expansion among CC animals involve representatives of the Pleosporales order, including members (wuc) of the Didymellaceae family. Interestingly, only two species of Basidiomycota were identified as over-represented in the feces of CC animals and both belong to the genus *Malassezia*, which congregates fungi traditionally involved with inflammatory processes, especially in the skin (*Malassezia japonica* and *Malassezia dermatis*). Animals from the SC group, on the other hand, presented expanded populations of Basidiomycota from the Cystobasidiomycetes class, including members of the Cystobasidiales order and Cystobasidiomycetaceae family, with emphasis on the species *Cystobasidium slooffiae*. Only three species of Ascomycota were identified as expanded among SC animals: *Cladosporium haloterians*, *Penicillium citrinum,* and one species (wuc) of *Aspergillus*.

**Figure 5:**
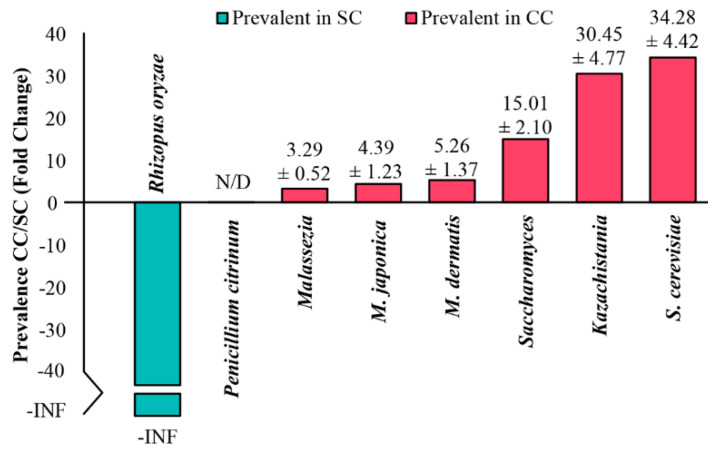
Relative quantification of different microorganisms in stool samples from SC and CC animals by qPCR. Equimolar samples of DNA extracted from stool samples obtained from each mouse were consolidated into two pools, representing SC and CC groups. Samples from these pools were then used in qPCR experiments, with primers previously described in the literature as specific to *Saccharomyces*, *Kazachstania,* and *Malassezia* genera, as well as to the species *Saccharomyces cerevisiae*, *Malassezia dermatis*, *Malassezia japonica*, *Rhizopus oryzae,* and *Penicillium citrinum*. The Ct values obtained for each of these taxa were normalized by the Ct obtained after amplifying the pooled DNAs with the primer pair used to amplify the fungal ITS1 amplicons. Next, the normalized Cts were used to calculate the relative prevalence of each genus/species in the CC pool, in relation to the SC pool (CC/SC). The relative quantification values for each taxon (Fold Change) are shown in the graph, as the mean ± standard deviation of three independent experiments (each performed in triplicate). The species *Rhizopus oryzae* was detected only in the DNA pool from SC animals, so its CC/SC ratio is represented in the graph as negative, to infinity (-INF). The species *Penicillium citrinum* was not detected (N/D) in any of the experiments, with either DNA pool.

Finally, to test the reliability of these results, the relative proportions of some of the taxa described above were evaluated in SC and CC animals, using real-time quantitative PCR (qPCR). As shown in Figure 5, these experiments confirmed the results obtained by the LEfSe analysis, showing a significant expansion of representatives of *Saccharomyces* and *Kazachstania* (as well as the species *S. cerevisiae*) within the gut of animals from the CC group (with more than one order of magnitude), in relation to SC. Representatives of the genus *Malassezia* (including *M. dermatis* and *M. japonica*) were also more represented in the pool of cachectic animals, although in smaller proportions (3 to 5×). Representatives of *Rhizopus oryzae*, on the other hand, were detected exclusively in the pool of SC animals, which also corroborates the results obtained from the LEfSe analysis. The only taxon that could not be amplified in these qPCR experiments was *Penicillium citrinum*, but we do not know if such negative results derive from under-representation of these fungi in the mycobiota from the two groups, or from problems regarding efficiency of the primers used in these experiments.

## 4. Discussion

Cachexia represents one of the most devastating clinical conditions that can occur with individuals affected by different types of cancer, since its high degree of morbidity results in significant reduction in the patients’ life quality. Moreover, treatments aimed at preventing or combating the development of CC often meet with limited success, since its onset and progression may be influenced by a variety of internal and external factors [1]. In this sense, the existence of correlations between the development of cachexia and intestinal dysbiosis, as observed in mice, suggests that manipulation of gut microbial communities may assist in the development of novel adjuvant approaches to alleviate symptoms in cachectic patients, or to prevent its onset in individuals at risk of developing such disorder. In this sense, the results presented herein demonstrate, for the first time, that intestinal dysbiosis associated with cachexia is not limited to changes in bacterial population, but also involves significant changes in the fungal population present in the gut of cachectic animals.

Analysis of relative ratios involving the main phyla of fungi are commonly performed to characterize fungal dysbiosis ratios (or DRs) that accompany the progression of other dysbiosis-associated diseases [23,44]. In this sense, DRs involving both Mucoromycota/Ascomycota and Mucoromycota/Basidiomycota populations increased in the CC animals, which display reduced proportions of Mucoromycota in their gut. Interestingly, a similar scenario has been reported by Mar Rodríguez et al. (2015) [45], who compared the gut mycobiota of lean and obese individuals. This similarity is noteworthy, since obesity and cachexia are both associated with imbalances in energy metabolism, affecting fat storages. In this study, the relative reduction in the population of *Mucor* spp. constituted the hallmark of the fungal dysbiosis verified in obese individuals, leading authors to suggest that the presence of chitosan in the cell wall of *Mucor* species could contribute to prevent obesity (a well-documented nutritional property of this polysaccharide) [45,46]. In this sense, *Rhyzopus oryzae*, the main Mucoromycota involved in our CC-associated dysbiosis, may also act as a potential chitosan source in the gut of healthy animals [47]. This information raises the interesting possibility of testing chitosan and some of its derivatives, as food supplements, in adjuvant protocols aimed at treating or preventing cachexia, which may enhance the current pharmaceutical and biomedical applications of this polysaccharide. Additionally, *R. oryzae* has been reported to produce substantial amounts of various antioxidants and organic acids, including gallic acid [48]. Interestingly, an extract of oil palm phenolics (OPP), containing 1500 ppm gallic acid equivalent, has been shown to inhibit tumorigenesis, by mediating G1/S phase cell cycle arrest, delay inflammatory responses and attenuate cachexia symptoms in tumor-bearing mice [49]. Gallic acid has also been shown to improve glucose tolerance and triglyceride concentration in obese mice [49] and suppress lipogenesis in humans, while concomitantly combating pro-inflammatory responses [50].

Specific dysbioses have also been associated with additional diseases and syndromes, including colorectal cancer (CRC), myalgic encephalomyelitis (ME) (also known as chronic fatigue syndrome, or CFS) and the two main forms inflammatory bowel disease (IBD): Chron’s disease (CD) and ulcerative colitis (UC) [51,52,53,54]. While the abovementioned diseases display alternative clinical manifestations, they all share common traits with obesity and cachexia, including increased permeability of the protective mucosal gut barrier and intestinal inflammation. Contrary to obesity and cachexia, however, CRC/ME/IDB/CD/UC do not affect adipose and muscular tissues and do not display gut dysbioses based on reduced ratios of Mucoromycota, but on increased ratios of specific Ascomycota groups. Interestingly, many of these Ascomycota are also expanded in the gut of CC animals, as verified by our LEfSe analysis (and confirmed by qPCR). For example, Luan et al. (2015) [55] demonstrated that members of the Sordariomycetes class (especially *Fusarium* and *Trichoderma*) are significantly enriched in rectal tissue samples from patients affected by CRC. Several species of *Fusarium* are recognized for causing infections involving both normal and immunosuppressed individuals and toxins produced by this group of fungi have already been shown to induce cell proliferation and affect different intestinal defense mechanisms [51]. Moreover, *Fusarium* toxins can severely reduce thickness of the intestinal mucus layer, increasing its permeability and allowing microorganisms present in the gut lumen to reach the intestinal wall, triggering the production of immunoglobulins and pro-inflammatory cytokines [56]. In fact, at least one *Fusarium* toxin, known as deoxynivalenol (DON) has already been correlated with the development of IBD [56], which is also in accord with the observations made by Liguori et al. (2016) [52], who detected expansion of Sordariomycetes in the inflamed intestinal mucosa of CD patients. In a recent publication, Chikina et al. (2020) [57] demonstrated that colon macrophages protect the intestinal epithelium from fungal toxins, preventing the induction of gut barrier dysfunction, which constitutes a hallmark of cachexia and other inflammatory bowel diseases, thus reinforcing the possibility that fungal toxins may play an important role during the development of CC [58].

Members of the Saccharomycetaceae family also showed increased population in the gut of patients affected by CD, with emphasis on *Candida glabrata*, a pathogen known to mediate inflammatory responses in the human intestine [44]. In this sense, our studies point to a significant expansion involving the populations of two species of Saccharomycetaceae, among the CC animals: *Saccharomyces cerevisiae* and *Kazachstania pintolopesii*. Interestingly, members of the *Kazachstania* genus show great phylogenetic proximity to *Candida* [59] and previous studies have even suggested that these two yeast genera could perform analogous functions in the intestinal microbial ecosystem [60]. Paradoxically, studies by Liguori et al. (2016) [52] identified an increased proportion of *Saccharomyces cerevisiae* in non-inflamed mucous membranes of healthy individuals, which led these authors to suggest that this yeast could play a beneficial role, preventing the development of CD. This proposal is in accord with results derived from other studies, involving both humans and mice, which suggested that *S. cerevisiae* could exert anti-inflammatory effects in host tissues, inducing the production of interleukin-10 [44,61].

However, Chiaro et al. (2017) [62] have argued otherwise, since the presence of *S. cerevisiae* exacerbated intestinal inflammation in a mice model of UC and increased the permeability of the protective intestinal mucosal barrier in these animals. In an independent study, Lamprinaki et al. (2017) [63] demonstrated that members of the Saccharomycetaceae family (particularly *S. cerevisiae* and *Kazachstania unispora*) are able to trigger inflammatory responses through the interaction of α-mannans, present in their cell walls, with the lectin dectin-2 of their host’s dendritic cells. Chiaro et al. (2017) [62] also demonstrated that the presence of *S. cerevisiae* seems to potentiate the production of metabolites capable of exacerbating inflammatory processes in their hosts, including increased production and accumulation of uric acid. Finally, anti-*S. cerevisiae* antibodies (ASCAs) are often found in patients with CD and UC, being used as serological markers for differential diagnosis between these two forms of IBD [64]. Taken together, these data reinforce the existence of a positive correlation between increased intestinal populations of *S. cerevisiae* and *K. pintolopesii* with the development of gut inflammatory responses and increased permeability of the protective mucosal barrier, along the intestine (two characteristics also observed during the development of cachexia).

Representatives of the genus *Malassezia*, the only Basidiomycota whose populations have been shown to expand in the gut of CC mice, have a long history of correlation with dysbioses associated with skin inflammation and dermatological diseases, since they are the main mycobiota components of the human and animal skin [65]. For example, Sokol et al. (2017) [53] found decreased Malasseziales populations in the intestine of patients affected by CD, while these same microorganisms were increased in the intestine of UC patients, suggesting that these fungi could be used as biomarkers to differentiate these two forms of IBD. More recently, Limon et al. (2019) [66] demonstrated that the species *Malassezia restricta* exacerbates symptoms of colitis in gnotobiotic mice and is present at a greater proportion in the colonic mycobiota of human subjects affected by CD [66]. Aykut et al. (2019) [67] also provided evidence that the population of *Malassezia* present in the digestive tract may be correlated with the development of other non-cutaneous pathologies, as representatives of this genus seem to compose a significant part of the microorganisms that invade the pancreatic duct, from the intestinal lumen, contributing to the development of ductal pancreatic adenocarcinoma (PDA), through activation of the complement cascade, via interaction with mannan-binding lectins (MBLs) [68]. More recently, increases in the relative proportion of *Malassezia* have also been found in the gut of CRC patients and of children showing autoimmunity against pancreatic beta cells, which later developed type 1 diabetes [23,69].

The cachexia-associated fungal dysbiosis associated with the LLC model is also characterized by additional fungal groups, whose relative proportions are decreased in CC animals (Figure 4). These include members of the Cystobasidiomycetes class (with emphasis on the species *Cystobasidium sloofiae*) and representatives (wuc) of the Sporidiobolaceae family. Although there are few reports regarding the involvement of these fungal groups with disease-associated dysbiosis, increased populations of Sporidiobolaceae have been detected in cervical mucosa lesions from HPV-infected women at low risk of malignancy, as well as in the intestine of mice submitted to diets rich in animal proteins [70]. Mar Rodríguez et al. (2015) [45], on the other hand, showed that the relative abundance of Cystobasidiomycetes was positively correlated with adiposity, while negatively correlated with the serum concentration of LDL cholesterol in obese and lean subjects.

As mentioned above, obesity-associated fungal dysbiosis essentially involves a reduced proportion of Mucoromycota, differing from the pattern observed in CRC/ME/IBD/UC/CD, which are mostly characterized by increased proportions of Ascomycota. Interestingly, the fungal dysbiosis of LLC-induced cachectic animals seems to display a unique pattern, presenting features observed both in obesity (reduced proportion of Mucoromycota) and CRC/ME/IBD/UC/CD, with increased proportions of different groups of Ascomycota (such as Sordariomycetes and Saccharomycetaceae), as well as *Malassezia*.

Finally, it is worth mentioning that the population analysis shown in Figure 4 allowed identification of four fungal species preferably present in the gut of SC animals, which can be grown under axenic laboratory conditions (*Rhizopus oryzae, Cystobasidium sloofiae, Penicillium citrinum,* and *Cladosporium halotolerans*). These fungi are natural candidates to be tested (individually, or in combinations) for their eventual roles as probiotic agents, aiming at the prevention/treatment of cachexia [48]. *Rhyzopus oryzae*, in particular, stands out as the most promising probiotic candidate, not only due to its capacity to produce bioactive substances, such as chitosan and gallic acid, but also to its historical use as a component of traditional oriental foods, which has granted it the status of a GRAS (generally recognized as safe) filamentous fungus, according to the US Food and Drug Administration (FDA) [48].

## 5. Conclusions

The results described herein indicate that fungal gut dysbiosis is also observed in a mouse model of cachexia, based on inoculation with Lewis lung carcinoma (LLC) cells. These results provide a reference profile for a cachexia-associated dysbiosis, employing one of the main animal models used to study this syndrome. Interestingly, the fungal gut dysbiosis associated with LLC-induced cachexia presents features observed in both obesity (reduced proportion of Mucoromycota) and CRC/ME/IBD (increased proportions of Sordariomycetes, Saccharomycetaceae and *Malassezia*). However, it is important to bear in mind that cachexia may be triggered by different factors, which affect the development and outcome of this syndrome in a variety of ways. Thus, it remains to be demonstrated if this particular dysbiosis profile is conserved in alternative cachexia models (e.g., induced by different tumor cells, or by heart/kidney chronic diseases), as well as when employing animals of varying ages, derived from different breeds and genetic backgrounds, or raised in alternative housing facilities, among other variables. Nonetheless, the LLC-related dysbiosis described herein provides a useful framework to test if mycobiota manipulation may be employed as an adjuvant therapy, aimed at treating and/or preventing the development of CC. In this sense, one species of Mucoromycota (*Rhyzopus oryzae*) stands out as a promising probiotic candidate to be tested in the LLC model of mouse cachexia.

## Appendix A

**Table A1:** Number of amplicons obtained after sequencing and preprocessing the ITS1 region of SC and CC animals.

Animals	Total of *Amplicons* Sequenced
**SC1**	109,236
**SC2**	66,436
**SC3**	120,142
**SC4**	135,404
**SC5**	163,377
**SC6**	144,145
**SC7**	133,910
**SC8**	153,817
**C** **C** **1**	155,586
**C** **C** **2**	141,331
**C** **C** **3**	121,782
**C** **C** **4**	124,370
**C** **C** **5**	104,937
**C** **C** **6**	137,693
**C** **C** **7**	130,312
**C** **C** **8**	120,530
**Total**	**1,036,541**
